# Sarco/Endoplasmic Reticulum Ca^2+^ ATPase 2 Activator Ameliorates Endothelial Dysfunction; Insulin Resistance in Diabetic Mice

**DOI:** 10.3390/cells11091488

**Published:** 2022-04-28

**Authors:** Toyokazu Kimura, Kazuki Kagami, Atsushi Sato, Ayumu Osaki, Kei Ito, Shunpei Horii, Takumi Toya, Nobuyuki Masaki, Risako Yasuda, Yuji Nagatomo, Takeshi Adachi

**Affiliations:** Department of Internal Medicine I, Division of Cardiovascular Medicine, National Defense Medical College, 3-2 Namiki, Tokorozawa 359-8513, Japan; oyotikuuyakim@gmail.com (T.K.); mirror.1028k@gmail.com (K.K.); atsushi19821005@yahoo.co.jp (A.S.); ayumu.osaki@gmail.com (A.O.); saintsilvesta@yahoo.co.jp (K.I.); shumhorii@outlook.jp (S.H.); con367@ndmc.ac.jp (T.T.); con320@ndmc.ac.jp (N.M.); cln302@ndmc.ac.jp (R.Y.)

**Keywords:** type 2 diabetes mellitus, sarco/endoplasmic reticulum Ca^2+^-ATPase2, endothelial function, skeletal muscle function, endoplasmic reticulum stress

## Abstract

**Background:** Sarco/endoplasmic reticulum Ca^2+^-ATPase2 (SERCA2) is impaired in various organs in animal models of diabetes. The purpose of this study was to test the effects of an allosteric SERCA2 activator (CDN1163) on glucose intolerance, hepatosteatosis, skeletal muscle function, and endothelial dysfunction in diabetic (*db*/*db*) mice. **Methods:** Either CDN1163 or vehicle was injected intraperitoneally into 16-week-old male control and *db*/*db* mice for 5 consecutive days. **Results:** SERCA2 protein expression was decreased in the aorta of *db*/*db* mice. In isometric tension measurements of aortic rings from *db*/*db* mice treated with CDN1163, acetylcholine (ACh)-induced relaxation was improved. In vivo intraperitoneal administrations of CDN 1163 also increased ACh-induced relaxation. Moreover, CDN1163 significantly decreased blood glucose in *db*/*db* mice at 60 and 120 min during a glucose tolerance test; it also decreased serum insulin levels, hepatosteatosis, and oxygen consumption in skeletal muscle during the early period of exercise in *db*/*db* mice. **Conclusions:** CDN1163 directly improved aortic endothelial dysfunction in *db*/*db* mice. Moreover, CDN1163 improved hepatosteatosis, skeletal muscle function, and insulin resistance in *db*/*db* mice. The activation of SERCA2 might be a strategy for the all the tissue expressed *SERCA2a* improvement of endothelial dysfunction and the target for the organs related to insulin resistance.

## 1. Introduction

Diabetes mellitus is considered a globally emergent disease because of the gradual increase in the number of patients who have it, and it is associated with a range of complications such as blindness, renal failure, and cardiovascular disease. In particular, the number of patients with type 2 diabetes mellitus (T2D) is constantly increasing, resulting in a considerable financial burden. According to the International Diabetes Federation Diabetes Atlas (8th edition), as of 2017, there are 425 million people with diabetes mellitus worldwide, which will rise to 629 million by 2045 [1].

T2D is characterized by chronic hyperglycemia, dyslipidemia, and insulin resistance [2,3]. The major problem caused by T2D is vascular complications, which are usually classified into two categories: microvascular (i.e., retinopathy, nephropathy, and neuropathy) and macrovascular complications (i.e., coronary heart disease, peripheral artery disease, and stroke). Clinical trials have shown that the intensive treatment of hyperglycemia is associated with a reduction in the development of microvascular complications [4,5]. On the other hand, clinical trials of T2D for anti-diabetic drugs were unsatisfied for macrovascular diseases. [5,6,7]. Lowering glucose levels might cause adverse events associated with hypoglycemia [8,9,10]. However, other factors, such as dyslipidemia, hepatosteatosis, and insulin resistance, might contribute to the development of macrovascular complications. Thus, new drug targets for insulin resistance for T2D have emerged as a strategy to improve insulin resistance and macrovascular diseases. Insulin resistance deteriorates glucose/lipid metabolisms, which induce endothelial dysfunction. Recently, we reported that endothelial insulin resistance impaired nitric oxide (NO) bioactivity resulting in vascular/heart failure [11]. Since insulin resistance is closely related to endothelial function, a new target for improving NO bioactivity might be a candidate.

Sarco/endoplasmic reticulum Ca^2+^-ATPase 2 (SERCA2), which is a membrane protein located in the endoplasmic reticulum (ER), uptakes Ca^2+^ from the cytoplasm into the ER lumen and maintains intracellular Ca^2+^ homeostasis [12]. SERCA2 dysfunction can increase intracellular Ca^2+^ levels and induce ER stress, which can lead to the development of T2D [13,14]. In recent years, SERCA2 has been found to be associated with the pathogenesis and cardiovascular complications of T2D [15,16,17]. Previously, we found that NO directly activated SERCA2 with S-glutathiolation at Cys674, leading to a decrease in intracellular calcium and arterial tonus [18,19].

Insulin resistance, oxidative stress, inflammation, and post-translational modifications, which are induced in T2D, can impair the expression and/or function of SERCA2 [13,17,20,21]. SERCA2 dysfunction has been observed in the progression of atherosclerosis, endothelial dysfunction, smooth muscle migration, dysfunction of angiogenesis, and impairment of heart function [18], which occur frequently in patients with diabetes.

Recently, it was reported that an allosteric SERCA2 activator, CDN1163 [22], ameliorated glucose metabolism and hepatosteatosis in obese (*ob*/*ob*) mice, a typical model of metabolic syndrome, by offsetting ER stress [23]. The data suggested that CDN1163 might be a strong candidate for the treatment of insulin resistance and glucose metabolism in patients with T2D. In this study, we investigated whether CDN1163 ameliorated glucose metabolism and vascular dysfunction in diabetic (*db*/*db*) mice.

## 2. Materials and Methods

### 2.1. Animals

Male control mice (*db*/+) [24] or *db*/*db* mice aged less than 12 weeks were obtained from Oriental Yeast (Tokyo, Japan) and fed a normal rodent diet (CLEA Japan, Inc., Tokyo, Japan). When aged 14–16 weeks, the control and *db*/*db* mice were injected intraperitoneally with either vehicle (10% DMSO, 10% Tween 80 in 0.9% NaCl (Wako Pure Chemical Corporation, Osaka, Japan)) or CDN1163 (100 mg/kg) for 5 consecutive days (days 0–4). CDN1163 was purchased from Neurodon (Crown Point, IN, USA). These mice were referred to as control+veh (control mice, vehicle), control+CDN (control mice, CDN1163), *db*/*db*+veh (*db*/*db* mice, vehicle), and *db*/*db*+CDN (*db*/*db* mice, CDN1163). The animals were maintained in a temperature-controlled facility on a 12-h light and 12-h dark cycle. This study was approved by the National Defense Medical College Institutional Animal Care and Use Committee (approval number: 15,074; number of mice used: 114).

### 2.2. Measurement of Body Weight and Food Intake

The body weight values were measured on day 0 before injection and day 7 in a non-fasted state. Their twenty-four-hour food intake was measured on days 7–9 by placing the animals in a clear cage with a food tray (Oxymax; Columbus Instruments, Columbus, OH, USA), and the daily mean value was defined as the food intake.

### 2.3. Measurement of Metabolites

The blood glucose levels were measured with a glucose-detection kit. Serum insulin levels were measured with an enzyme-linked immunosorbent assay kit, and the serum levels of aspartate aminotransferase (AST), alanine aminotransferase (ALT), total cholesterol (TC), and triglyceride (TG) were measured with enzymatic assays. All kits and assays were obtained from Wako Pure Chemical Corporation (Osaka, Japan). An oral glucose tolerance test (OGTT) was performed after fasting the mice for 14 h. Blood was collected from the tail vein at 0, 15, 30, 60, and 120 min after 1.5 mg/g body weight of D-glucose was administered orally to the mice [25]. Serum insulin levels were measured using blood samples at 0 and 120 min of the OGTT, and insulin secretion was measured at 0 and 120 min.

### 2.4. Western Blotting Analysis

Tissues were excised and homogenized on ice in a homogenization buffer (20 mmol/L Tris-HCl, 150 mmol/L NaCl, 1 mmol/L Na_2_EDTA, 1 mmol/L EGTA, 1% NP-40, 2.5 mmol/L sodium pyrophosphate, 1 mmol/L monoglycerophosphate, 1 mmol/L Na_2_VO_4_, pH 7.4) containing a 1 mmol/L phenylmethylsulfonyl fluoride and protease inhibitor cocktail. All reagents were obtained from Wako Pure Chemical Corporation (Osaka, Japan). The homogenates were centrifuged at 13,000× *g* for 20 min. A Bradford protein assay (Bio-Rad laboratories, CA, USA) was performed to measure protein concentration with bovine serum albumin as a standard [26]. For the Western Blotting of SERCA2, the samples were not incubated at 95 °C to avoid SERCA2 protein aggregation. The samples were run on a sodium dodecyl sulfate-polyacrylamide gel and transferred onto a polyvinylidene difluoride membrane. After blocking, the gel was incubated overnight at 4 °C with a primary anti-SERCA2 antibody (Santa Cruz, Dallas, TX, USA and Thermo Fisher Scientific, Waltham, MA, USA).

### 2.5. Hematoxylin and Eosin Staining

The mice were sacrificed with triple anesthesia containing 0.3 mg/kg medetomidine, 4.0 mg/kg midazolam, and 5.0 mg/kg butorphanol [27] and perfused with 0.9% saline followed by 4% paraformaldehyde. The livers were excised and fixed in 10% formalin overnight, embedded in paraffin, and sectioned (3 μm thick). All samples were stained with hematoxylin and eosin. Images were obtained with BZ-X710 (Keyence, Osaka, Japan). The lipid droplet area divided by the tissue area excluding the luminal structures was evaluated.

### 2.6. Metabolic Chamber Experiments

The metabolic parameters were measured using an indirect open-circuit calorimeter (Oxymax; Columbus Instruments, Columbus, OH, USA), as described previously, with some modifications [28]. The mice were placed in clear chambers and could move freely for 24 h in a 12 h light and 12 h dark cycle. A water bottle was connected to each chamber, and a food tray was also placed there. The flow rate of room air through the chamber was 0.5 L/min. Exhaled gases were passed through the O_2_ and CO_2_ sensors and measured every 10 min to calculate oxygen consumption (VO_2_) and carbon dioxide production (VCO_2_). The gas sensors were calibrated using a standard gas with known concentrations of N_2_, O_2_, and CO_2_ (Koatsu Gas Kogyo, Shiga, Japan) before each experiment. The respiratory exchange ratio (RER) was calculated as VCO_2_ divided by VO_2_. The mice were placed in the chamber for 2 days for acclimation before the experiments.

### 2.7. Exercise Capacity with a Treadmill Test

A treadmill test was conducted using a Metabolic Modular Treadmill (Columbus Instruments, Columbus, OH, USA), as described previously, with some modifications [29]. For acclimation to the exercise, the mice were allowed to practice for 2 days before the experiment by being placed in the chamber with the treadmill at speed 0 m/min for the first 5 min, then the shock grid was activated. After that, the mice walked at a speed of 4 m/min for the next 10 min. In the treadmill test, after being placed on the stationary treadmill for 5 min, the speed of the treadmill was increased to 2 m/min for 5 min. Then, the speed was increased to 3 m/min for 2 min, after which the speed was increased by 1 m/min every 2 min thereafter. The test was continued until the mice remained in contact with the shock grid for more than 5 s. We measured running distance, VO_2_, and VCO_2,_ and also calculated oxygen consumption at the modified ventilatory threshold (mVT), VO_2mVT_. VT is generally defined as the exercise intensity at which VCO_2_ increases sharply during exercise. VO_2_ continues to increase in proportion to the load, but VCO_2_ increases rapidly at one point. However, in our *db*/*db* mice, VO_2_ did not increase continually. Therefore, mVT was defined as the exercise intensity at which VCO_2_/VO_2_ (i.e., RER) increased sharply during exercise. We plotted three graphs with time as the horizontal axis and VO_2_, VCO_2_, or RER as the vertical axis; VO_2mVT_ was calculated using these graphs.

### 2.8. Isometric Tension Measurement with an Organ Chamber

Isometric tension measurement was performed as described previously [30]. The mice were sacrificed with triple anesthesia containing 0.3 mg/kg medetomidine, 4.0 mg/kg midazolam, and 5.0 mg/kg butorphanol [27]. The thoracic aorta was excised, and adherent fat was removed carefully to avoid damaging the endothelium and then cut into 2.5–3 mm rings. The aorta was mounted in an organ bath filled with Krebs–Ringer bicarbonate solution (118.3 mmol/L NaCl, 4.7 mmol/L KCl, 2.5 mmol/L CaCl_2_, 1.2 mmol/L MgSO_4_, 1.2 mmol/L KH_2_PO_4_, 25 mmol/L NaHCO_3_, 5.5 mmol/L D-glucose) aerated with 95% O_2_ and 5% CO_2_ at 37 °C. The aorta was attached to a force transducer, and isometric tension was recorded. Initially, a 1 g pre-tension was applied to all rings for 1 h. All rings were immersed in a 60 mmol/L KCl solution to confirm contraction. After washout, L-phenylephrine (10^−6.5^ mol/L) was added to each chamber to contract all rings, and, after the contraction curve reached a plateau, the rings were exposed to increasing concentrations of ACh (10^−9^ to 10^−5^ mol/L) or sodium nitroprusside (SNP; 10^−9^ to 10^−5^ mol/L). In ex vivo experiments, the rings were pretreated with either DMSO (5 μL) or CDN1163 (1 μmol/L) for 15 min before precontraction with L-phenylephrine. After this pretreatment, we performed the same procedures as in the in vivo experiments.

### 2.9. Statistical Analysis

Data are expressed as the mean ± standard error of the mean (SEM). The OGTT and vascular relaxation were evaluated by two-way ANOVA with repeated measures followed by a *post hoc* test with Bonferroni’s correction for multiple comparisons. The treadmill test was evaluated by two-way ANOVA with repeated measures, and a *t* test was used to compare individual time points [31]. The other data were evaluated either by a one-way ANOVA followed by a post hoc test with Bonferroni’s or Tukey’s correction or by the Mann–Whitney *U* test. All statistical analysis were performed with GraphPad Prism Software version 7.03 (GraphPad Software, La Jolla, CA, USA). A value of *p* < 0.05 was considered to be statistically significant.

## 3. Results

### 3.1. Decreased SERCA2 Expression in the Aorta of db/db Mice

**Figure 1:**
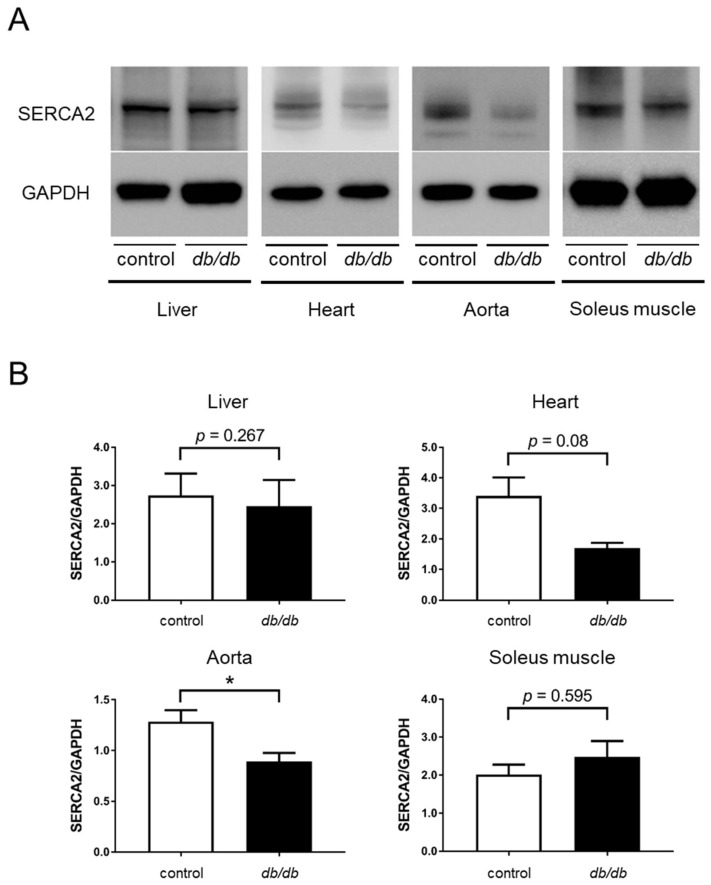
SERCA2 expression in the tissues of *db*/*db* mice compared with control mice. (**A**) SERCA2 expression in liver, heart, aorta, and soleus muscle by western blotting. (**B**) Relative quantification of SERCA2 expression to control mice (*n* = 4 in heart and soleus muscle and n = 6 in liver and aorta for each group). Error bars represent SEM. * *p* < 0.05 vs. control. SERCA2, sarco/endoplasmic reticulum Ca^2+^-ATPase 2; GAPDH, glyveraldehehyde-3-phosphate.

First, we examined SERCA2 protein expression in homogenates from the liver, heart, aorta, and soleus muscle of *db*/*db* mice. The soleus muscle is slow-twitch muscle and contains more SERCA2 proteins compared with fast-twitch muscles. There was no significant difference in the expression of SERCA2 protein in the liver and soleus muscle, it had a tendency to decrease in the heart and was significantly decreased in the aorta from *db*/*db* mice (Figure 1).

### 3.2. Decreased Body Weight and Ameliorated OGTT with CDN1163 in db/db Mice

**Figure 2:**
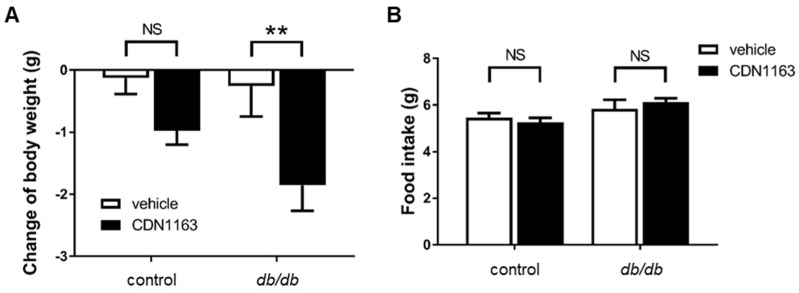
CDN1163 decreases the body weight of *db*/*db* mice. (**A**) Change of body weight between day 0 and day 7 (n = 11 for each group). (**B**) Twenty-four-hour food intake was measured using a food tray in a clear cage in which the mice were placed individually (control+veh, n = 8; control+CDN, n = 7; *db*/*db*+veh, n = 5; *db*/*db*+CDN, n = 6). Error bars represent SEM. ** *p* < 0.01 vs. *db*/*db*+veh. CDN indicates CDN1163; veh, vehicle; NS, not significant.

We measured the body weight of the mice before and at seven days after the administration of CDN1163 or vehicle. There was significant weight loss in the *db*/*db*+CDN group compared with the *db*/*db*+veh group (Figure 2A); however, there was no significant difference in food intake (Figure 2B). CDN1163 did not decrease the body weight of the control mice.

**Figure 3:**
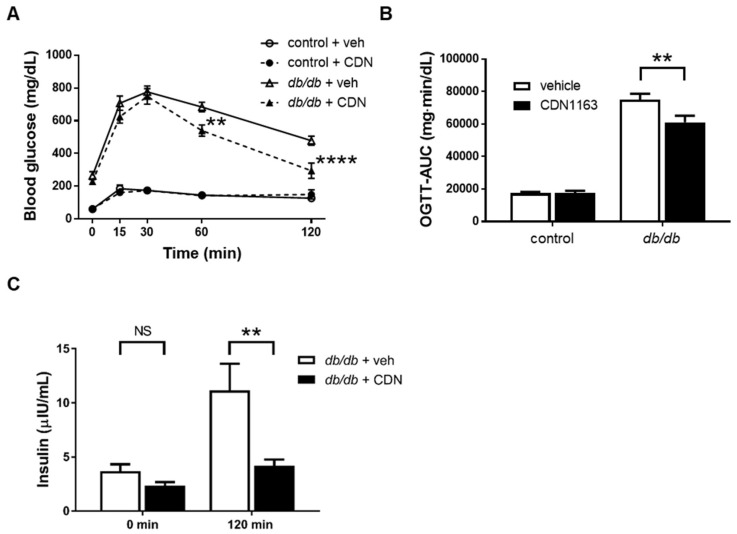
CDN1163 ameliorates glucose tolerance in *db*/*db* mice. (**A**,**B**) OGTT and OGTT-AUC for glucose levels. OGTT results are expressed as mean blood glucose concentration ± SEM (control+veh, n = 7; control+CDN, n = 5; *db*/*db*+veh, n = 7; *db*/*db*+CDN, n = 5). (**C**) Serum insulin levels were measured at 0 min and 120 min after glucose administration (n = 10 for each group). Error bars represent SEM. ** *p* < 0.01, **** *p* < 0.0001 vs. *db*/*db*+veh. AUC, area under the curve; CDN, CDN1163; NS, not significant; OGTT, oral glucose tolerance test; veh, vehicle.

To evaluate glucose metabolism, an OGTT was performed. There was a significant decrease in the blood glucose levels of the *db*/*db*+CDN group at 60 and 120 min after the oral administration of D-glucose compared with the *db*/*db*+veh group, and there was no significant difference between the control+veh and control+CDN groups (Figure 3A,B). We also measured the serum insulin concentration at 0 and 120 min after D-glucose administration, which decreased significantly at 120 min in the *db*/*db*+CDN group compared with the *db*/*db*+veh group (Figure 3C). These data indicate that CDN1163 improved glucose tolerance by improving insulin sensitivity, not by increasing insulin secretion.

### 3.3. Amelioration of Hepatosteatosis with CDN1163

**Figure 4:**
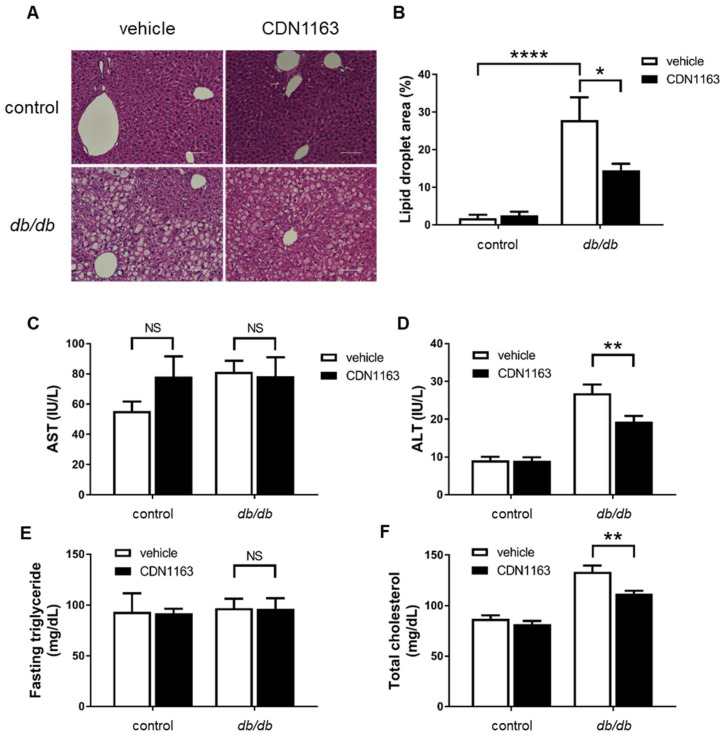
CDN1163 improves hepatosteatosis in *db*/*db* mice. (**A**) Hematoxylin and eosin staining of liver sections for control and *db*/*db* mice with either vehicle or CDM1163. Scale bars, 100 μm. (**B**) Percentage of lipid droplet area. The value was calculated as lipid droplet area divided by tissue area excluding luminal structures (n = 5 for each group). (**C**) Serum AST levels (n = 9 for each group). (**D**) Serum ALT levels (n = 9 for each group). (**E**) Serum fasted TG levels (Ctrl+veh, n = 4; Ctrl+CDN, n = 4; Db+veh, n = 5; Db+CDN, n = 4). (**F**) Serum TC levels (n = 9 for each group). Error bars represent SEM. * *p* < 0.05, ** *p* < 0.01, **** *p* < 0.0001 vs. Db+veh. AST, aspartate aminotransferase; ALT, alanine aminotransferase; Db, *db*/*db* mice; TG, triglyceride; TC, total cholesterol; NS, not significant.

Although there was no difference in the expression of SERCA2 protein in the liver between the control+veh and *db*/*db*+veh groups, we evaluated hepatic function and the lipid profile using histology and by measuring AST, ALT, fasting TG, and TC, which are essential for glucose metabolism. Histologically, the percentage of lipid droplet area increased significantly in the *db*/*db*+veh group compared with the control+veh group, and its percentage decreased significantly in the *db*/*db*+CDN group compared with the *db*/*db*+veh group (Figure 4A,B). There was no significant difference in AST levels between the *db*/*db*+veh and *db*/*db*+CDN groups, whereas ALT levels were lower in the *db*/*db*+CDN group compared with the *db*/*db*+veh group (Figure 4C,D). TC levels were lower in the *db*/*db*+CDN group compared with the *db*/*db*+veh group, but fasting TG levels were not affected (Figure 4E,F).

### 3.4. Increased VO_2_ during Non-Exercise Load and VO_2mVT_ with CDN1163 in the Treadmill Test of db/db Mice

**Figure 5:**
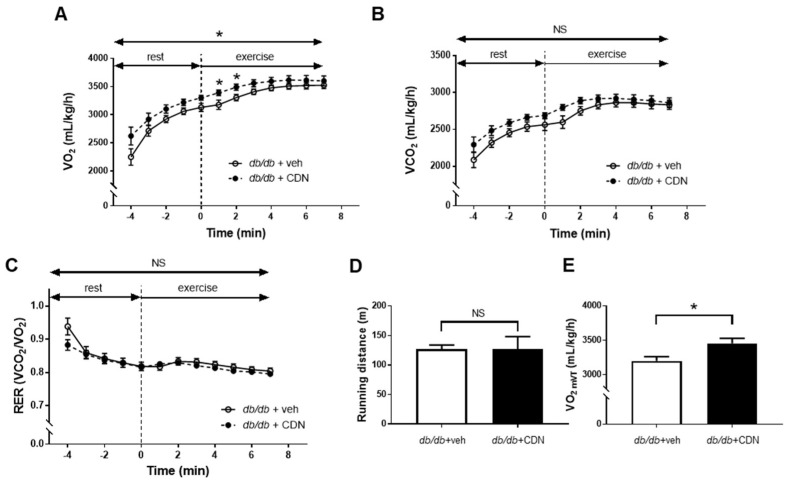
CDN1163 increases VO_2_ of *db*/*db* mice during the early phase of the treadmill test. (**A**–**C**) Measurement of VO_2_ (**A**), VCO_2_ (**B**), and RER (**C**) during 5 min of rest (−5–0 min) and the first 7 min of exercise (0–7 min). RER was calculated using VO_2_ and VCO_2_ (n = 11 for each). Two-way analysis of variance was used for the comparison of VO_2_, VCO_2_, and RER in both groups during the entire period, and the *t*-test was used for the comparison of individual time points. (**D**) Measurement of running distance (n = 11 for each). (**E**) Measurement of VO_2mVT_ (n = 11 for each). Modified VT (mVT) was defined as exercise intensity at which VCO_2_/VO_2_ (RER) started to increase sharply during exercise, and VO_2mVT_ was defined as VO_2_ at the mVT. Error bars represent SEM. * *p* < 0.05 vs. Db+veh. CDN, CDN1163; Db, *db*/*db* mice; NS, not significant; RER, respiratory exchange ratio; VCO_2_, carbon dioxide production; veh, vehicle; VO_2_, oxygen consumption; VO_2mVT_, oxygen consumption at modified VT; VT, ventilatory threshold.

As described previously, the expression of SERCA2 protein in the soleus muscle did not decrease in *db*/*db* mice compared with control mice. However, the skeletal muscle is well known as a major tissue for extracting glucose from the blood, especially during exercise. Thus, we evaluated the effect of CDN1163 on skeletal muscle function with metabolic chamber experiments. During non-exercise stress, there was no significant difference in VO_2_ and VCO_2_ between the *db*/*db*+veh and *db*/*db*+CDN groups (data not shown). RER increased considerably in the *db*/*db*+CDN group compared with the *db*/*db*+veh group, although the difference did not reach statistical significance (*p* = 0.057, data not shown). This implies that CDN1163 might increase the utilization of glucose in *db*/*db* mice. In the treadmill test, there was no significant difference in VCO_2_, RER, and running distance between the *db*/*db*+veh and *db*/*db*+CDN groups (Figure 5B–D). However, CDN1163 significantly increased VO_2_ at the early time point after starting exercise and VO_2mVT_ in *db*/*db* mice (Figure 5A,E). These data suggest that CDN1163 improved mitochondrial respiration during modest intensity exercise with a higher endurance capacity in aerobic metabolism.

### 3.5. Restored Endothelium-Dependent Relaxation of Aortic Rings from db/db mice with CDN1163

**Figure 6:**
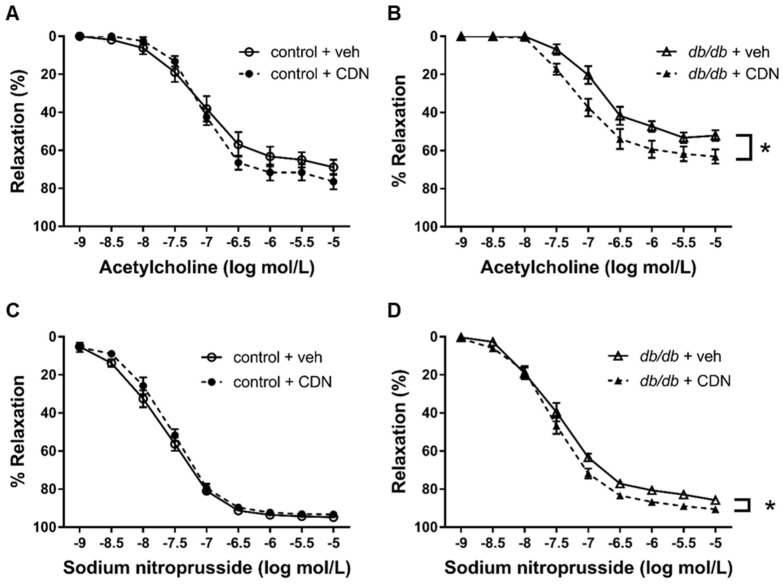
CDN1163 increases endothelium-dependent and endothelium-independent relaxation of *db*/*db* mice in in vivo experiments. (**A**,**B**) Vascular relaxation of aortic rings isolated from control mice (**A**) (Ctrl+veh, n = 11; Ctrl+CDN, n = 9) or *db*/*db* mice (**B**) (Db+veh, n = 10; Db+CDN, n = 11) with ACh. (**C**,**D**) Vascular relaxation of aortic rings isolated from control mice (**C**) (n = 9 for each group) or *db*/*db* mice (**D**) (n = 9 for each group) with SNP. Results of relaxation are expressed as percentage changes in the steady-state level of contraction with 10^−6.5^ mol/L L-phenylephrine. Error bars represent SEM. * *p* < 0.05 vs. Db+veh. CDN, CDN1163; Ctrl, control mice; Db, *db*/*db* mice; veh, vehicle; SNP, sodium nitroprusside.

In general, exercise stress increases blood flow considerably in the skeletal muscle by shear stress-induced endothelium-dependent relaxation. Endothelial dysfunction in the aortic rings of *db*/*db* mice has been reported previously [32]. SERCA2 plays a crucial role in NO-induced relaxation; thus, we hypothesized that the increase in skeletal muscle metabolism by CDN1163 might be due to an improvement of NO-induced relaxation. Next, we performed organ chamber experiments with ACh to evaluate if CDN1163 might improve the endothelium-dependent relaxation of the aortic rings of *db*/*db* mice. First, we tested the effects of in vivo treatment with CDN1163 on the ACh-induced relaxation of the aortic rings. ACh-induced relaxation was impaired in aortic rings from the *db*/*db*+veh group compared with the control+veh group; however, CDN1163 restored ACh-induced relaxation in the aortic rings of the *db*/*db* mice, although it had little effect on the aortic rings of the control mice (Figure 6A,B). Furthermore, CDN1163 increased the SNP-induced relaxation of the aortic rings of *db*/*db* mice (Figure 6C,D). These data indicate that in vivo treatment with CDN1163 increased endothelium-dependent relaxation and ameliorated smooth muscle response to NO donor from *db*/*db* mice.

**Figure 7:**
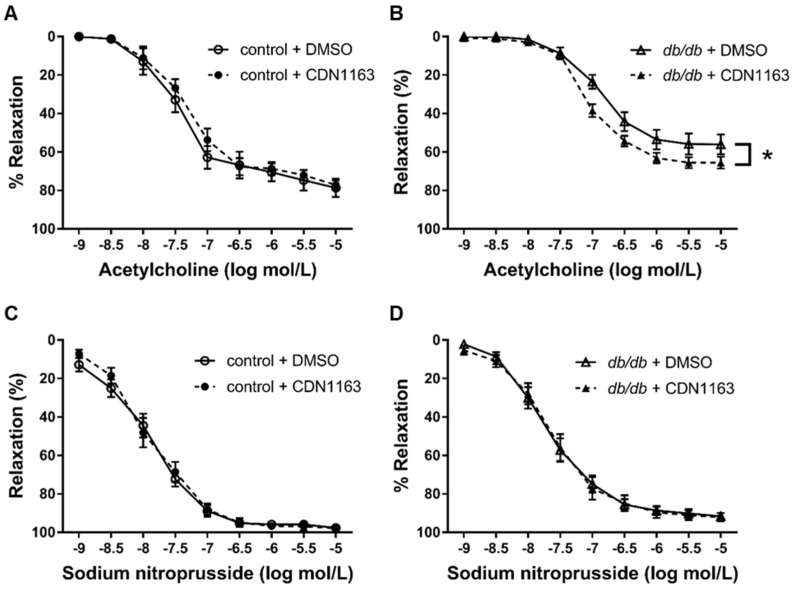
CDN1163 increases endothelium-dependent function in *db*/*db* mice in ex vivo experiments. All experiments were conducted on the condition that either DMSO (5 μL) or CDN1163 (1 μM, 5 μL) was added to the bath for 15 min before L-phenylephrine precontraction. (**A**,**B**) Vascular relaxation of aortic rings isolated from control mice (**A**) (n = 8 for each group) or *db*/*db* mice (**B**) (n = 12 for each group) with ACh. (**C**,**D**) Vascular relaxation of aortic rings isolated from control mice (**C**) (n = 8 for each group) or *db*/*db* mice (**D**) (n = 12 for each group) with SNP. Results of relaxation are expressed as percentage changes in the steady-state level of contraction with 10^−6.5^ mol/L L-phenylephrine. Error bars represent SEM. * *p* < 0.05 vs. Db+DMSO. CDN, CDN1163; Ctrl, control mice; Db, *db*/*db* mice; veh, vehicle; SNP, sodium nitroprusside.

To assess the direct effect of CDN1163 on the aorta, we performed organ chamber experiments with ex vivo treatment of CDN1163. CDN1163 was added to the Krebs–Ringer solution in the organ chamber for 15 min before contraction with L-phenylephrine. Pretreatment with CDN1163 also increased the endothelium-dependent relaxation of the aortic rings of *db*/*db* mice, although it had no significant effect on the aortic rings of control mice (Figure 7A,B). In this protocol, pretreatment with CDN1163 had no effect on the SNP-induced relaxation of the aortic rings of *db*/*db* and control mice (Figure 7C,D). The data suggest that CDN1163 has a direct effect on endothelium-dependent relaxation in *db*/*db* mice.

## 4. Discussion

T2D is characterized by glucose intolerance, dyslipidemia, and insulin resistance. Although various glucose-lowering drugs have been developed, there are a limited number of drugs that are effective for macrovascular complications. In the present study, we employed *db*/*db* mice, a typical murine model of T2D, and investigated the effects of an allosteric SERCA2 activator, CDN1163, on metabolism and endothelial function. Initially, we examined the metabolic effects of CDN1163. In spite of it having no effect on food intake, CDN1163 modestly decreased the body weight of the *db*/*db* mice, which was not observed in the control mice. CDN1163 did not change either the blood glucose or the insulin levels after fasting and before the oral administration of D-glucose in an OGTT. However, CDN1163 decreased the blood glucose levels at 60 and 120 min in the OGTT, and the insulin levels were decreased at 120 min in *db*/*db* mice. Fasting blood glucose levels are mostly dependent on gluconeogenesis, mainly from the liver [33]. On the other hand, lowering blood glucose levels in the latter phase is dependent on glucose uptake, which is mainly dependent on the function of the skeletal muscle and increased blood flow through arterioles by insulin [34,35]. Therefore, these data indicate that CDN1163 had a minimal effect on gluconeogenesis but increased the uptake of glucose by skeletal muscle. CDN1163 also decreased serum insulin levels at 120 min only in *db*/*db* mice. From these data, we considered that CDN1163 ameliorated glucose metabolism not by improving the secretion of insulin from β-cells, but by improving the insulin sensitivity of peripheral organs such as skeletal muscle. Among the large number of anti-diabetic drugs, metformin, which improves insulin resistance, is the current first choice drug for the treatment of T2D, because it can improve the outcome of macrovascular complications [36]. The insulin-sensitizing effects of SERCA2 are fascinating as a drug target for diabetes with the aim of improving insulin sensitivity.

Kang et al. reported the beneficial effects of CDN1163 in *ob/ob* mice, which is a typical murine model of obesity [23]. CDN1163 improved glucose tolerance and decreased blood glucose levels in the absence of a reduction in food intake in *ob/ob* mice. On the other hand, a decrease in blood glucose was not observed in lean control mice. These effects were compatible with our results, indicating that the effect of CDN1163 could be seen exclusively in *ob/ob* or *db*/*db* mice, and that CDN1163 was unlikely to trigger hypoglycemia.

We observed favorable effects of CDN1163 on hepatosteatosis in *db*/*db* mice. Although AST levels were not decreased, ALT levels, which are considered to be the more dominant enzyme in hepatosteatosis, were decreased by CDN1163 in *db*/*db* mice. TC levels decreased slightly, but fasting TG levels were not changed by CDN1163 in *db*/*db* mice. These data suggest that the short-term administration of CDN1163 contributed to a modest amelioration of hepatosteatosis. Considering that fasting blood glucose levels were not decreased by CDN1163, its effect might be smaller in *db*/*db* mice than in *ob/ob* mice [23]. In general, hepatosteatosis is more prominent in obesity than in T2D; thus, we might observe a difference in drug potency on hepatosteatosis simply due to the different models. Compared with *ob/ob* mice, endothelial and muscle dysfunction is more prominent in *db*/*db* mice. We reported that liver-specific ERK2 KO mice deteriorated hepatosteatosis by ER stress with decreases in hepatic SERCA2 expression, resulting in worsening insulin resistance and endothelial dysfunction [30]. We prepared to improve NO bioactivity by genetic modification in the endothelium and observed whether insulin resistance and hepatosteatosis were improved (ongoing study). We have interest in the close relationship among insulin resistance, hepatosteatosis, and endothelial dysfunction in diabetes. Our results from the OGTT also indicated that increased blood flow was related to the favorable effects of CDN1163. To study the effects of CDN1163 on muscle function, we originally investigated the physiological effects of CDN1163 on skeletal muscle and endothelial function, which are generally impaired in diabetes.

In this study, we examined how the expression of the SERCA2 protein changes in the liver, heart, aorta, and soleus muscle of *db*/*db* mice compared with control mice. Shah and Brownlee showed that hyperglycemia provided uridine diphosphate-*N*-acetylglucosamine, which is a substrate for the enzyme *O*-linked *N*-acetylglucosamine transferase, and *O*-linked *N*-acetylglucosamine transferase modified transcription complex factors regulating the expression of SERCA2, thereby reducing its levels [17]. Thus, hyperglycemia in diabetes can reduce the expression of the SERCA2 protein. In *db*/*db* mice, we observed that the reduced expression of the SERCA2 protein in the aorta had tendency to be decreased in the heart and not in the liver and soleus muscle compared with control mice of a similar age (Figure 1). All the tissue expressed SERCA2a and SERCA2b. SERCA2a was predominantly expressed in the heart and the skeletal muscle, and SERCA2b was predominantly expressed in the aorta and liver [37]. Thus, subtypes could not be reasons for the different expression in each tissue. Oxidative stress, such as hydrogen peroxide and peroxynitrite, which is produced in diabetes, can also decrease the expression and function of SERCA2 [38,39]. Further studies are required to clarify the complex regulations of SERCA2 expression in diabetes. As mentioned above, insulin resistance in the latter phase of the OGTT is dependent on skeletal muscle function and blood flow regulated by endothelial function. Hence, we examined how CDN1163 affected skeletal muscle and endothelial function.

We focused on changes in oxygen consumption by CDN1163, which is mainly represented as the mitochondrial function of the muscles, especially during exercise [40]. In the non-exercise state, CDN1163 did not change VO_2_ or RER of *db*/*db* mice compared with vehicle-treated mice (*p* = 0.057, data not shown). RER increases if the glucose metabolism is accelerated compared with lipid metabolism as an energy source. Due to the sedentary nature of *db*/*db* mice, we applied exercise to these animals to increase mitochondrial respiration in the muscle. In the treadmill test, CDN1163 significantly increased VO_2_ during the early phase of exercise and VO_2mVT_ in *db*/*db* mice. Previous studies reported that the O_2_ uptake response during low to moderate exercise was impaired in T2D, which might be due to the impairment of blood flow in the skeletal muscle [41,42]. The increase of VO_2_ in the early phase indicated that CDN1163 improved mitochondrial O_2_ uptake in *db*/*db* mice. When the exercise load is low, there is sufficient ATP to produce energy by utilizing glucose or fat with oxygen (aerobic metabolism) in mitochondria. In this phase, CDN1163 might increase the consumption of ATP by SERCA2 and improve mitochondrial oxygen consumption in the skeletal muscle, resulting in increased VO_2_. On the other hand, as the load increases during exercise, the consumption of ATP will further increase and be exhausted. In this state, the production of ATP without oxygen will be activated, the level of lactic acid is elevated (anaerobic metabolism), and the production of CO_2_ is increased by metabolizing lactic acid. This state is represented by VT. CDN1163 increased the VO_2mVT_ of *db*/*db* mice, indicating that CDN1163 increased oxygen consumption at the point when aerobic metabolism switched to anaerobic metabolism. These data suggested that CDN1163 improved skeletal muscle function and mitochondrial metabolism, which was impaired in *db*/*db* mice. Skeletal muscle metabolism and function was relied on by the blood flow during exercise. Sheer stress in exercise evoked endothelial-dependent, NO-induced relaxation in arteriole and augment blood flow. Thus, the enhanced NO-induced relaxation with CDN1163 could improve the skeletal muscle function. A recent report showed that CDN1163 directly improved myotube metabolism and function [43]. Thus, CDN1163 improved the skeletal muscle function by these two mechanisms. Next, we tested the effects of CDN1163 on endothelial function.

Our analysis with isometric tension measurement revealed that CDN1163 restored ACh-induced relaxation in *db*/*db* mice with in vivo and ex vivo treatment. In addition, CDN1163 also ameliorated SNP-induced relaxation in *db*/*db* mice with in vivo treatment, although such an effect was not seen with ex vivo treatment. These results indicate that CDN1163 had favorable effects on endothelium-dependent relaxation. NO released from endothelial NO synthase mainly activates guanylate cyclase and increases the levels of cyclic guanosine monophosphate (cGMP). cGMP activates cGMP-dependent protein kinase G, regulates myosin light chain phosphatase, and relaxes vessels [44]. On the other hand, NO activates SERCA2 in aortic smooth muscle in a partially cGMP-independent manner [19,20], which decreases intracellular Ca^2+^ and vessel relaxation. This mechanism has a role in S-glutathiolation at Cys674 on SERCA2 and atherosclerosis, aging, and heart failure, while, in diabetes, the cysteine residue is oxidized and impairs the mechanism [45]. The regulation of SERCA2 by NO in endothelial cells is also important for endothelial function and angiogenesis [46]. Many diseases decreased the aortic SERCA2 activity and NO-induced relaxation. Antioxidants improved SERCA2 activity and increased NO-induced relaxation [18,19]. Thus, we considered that the activation of SERCA2 with CDN1163 could improve NO-induced relaxation in *db*/*db* mice. We observed that in vivo treatment with CDN1163 restored SNP-induced vessel relaxation in *db*/*db* mice, suggesting that CDN1163 improved not only the endothelial function, but also the smooth muscle response to NO donor. We did not observe effects on the smooth muscle in our ex vivo experiments, potentially due to the period of administration or different sensitivity between the endothelium and smooth muscle. The restoration of the endothelial function observed following the systemic or direct administration of CDN1163 to the aortic rings might at least partially explain its favorable metabolic effects and the improvement of muscle function. These results indicate the important relationship between a decreased endothelial function and the SERCA2 function in diabetes.

**Figure 8:**
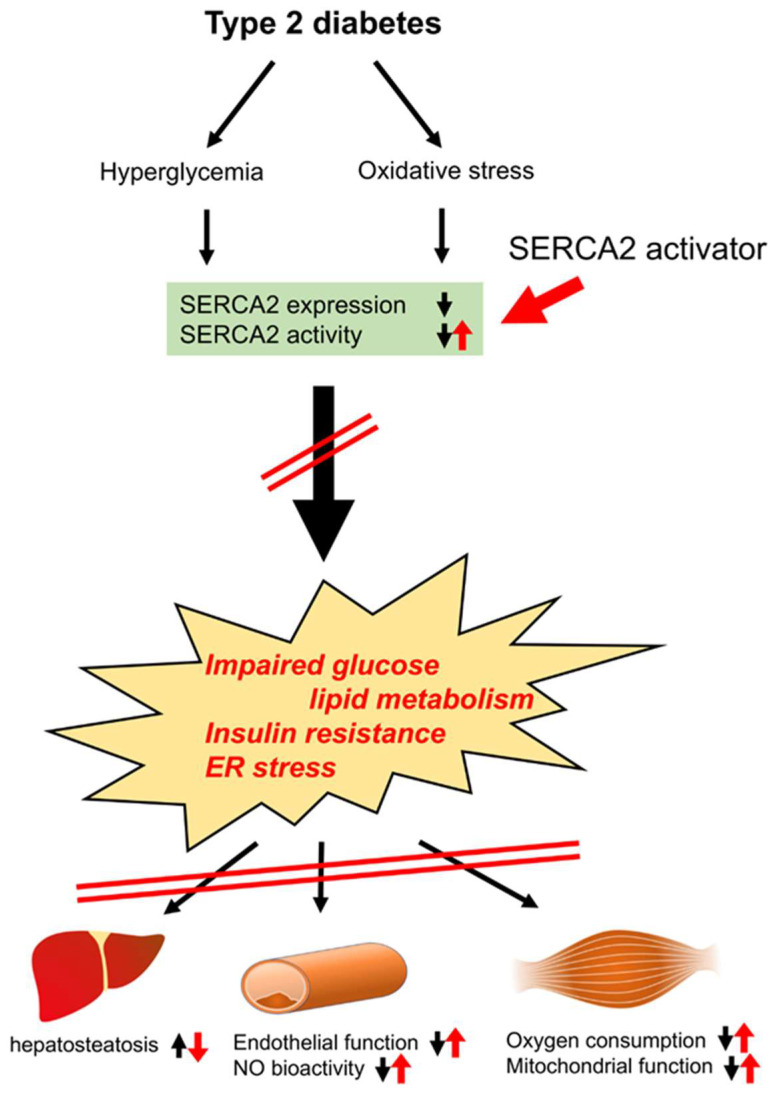
Mechanisms by which SERCA2 activator (CDN1163) improves the function of the liver, vascular tissue, and skeletal muscle. Hyperglycemia and oxidative stress induced in type 2 diabetes decrease SERCA2 expression and activity. CDN1163 ameliorates impaired glucose lipid metabolism, insulin resistance, and ER stress, then improves hepatosteatosis in the liver, endothelial dysfunction and NO bioactivity in vessels, and oxygen consumption and mitochondrial dysfunction in skeletal muscle. ER, endoplasmic reticulum; SERCA2, sarco/endoplasmic reticulum Ca^2+^-ATPase 2; NO, nitric oxide.

As endothelial dysfunction and insulin resistance are closely associated with macrovascular complications in patients with T2D [47], their improvement with a SERCA2 activator is an appealing strategy for the treatment of vascular complications. Insulin resistance is characterized by the impairment of the insulin receptor substrate/phosphatidylinositol 3-kinase/protein kinase B (Akt) pathway. In endothelial cells, Akt phosphorylates endothelial NO synthase at Ser1177 [48], and endothelial NO synthase can also be activated through the intracellular Ca^2+^ binding Ca^2+^/calmodulin complex. As CDN1163 improved endothelial function and insulin sensitivity, the activation of SERCA2 may improve both of them, which are impaired in T2D. It is well known that ER stress is one of the causes of T2D [49,50], and SERCA2 dysfunction triggers ER stress. Due to the induction of ER stress, various organs or tissues may fail to work normally. ER stress is associated with hepatosteatosis in the liver [51,52], endothelial dysfunction in the aorta [32], reduced insulin secretion from β-cells [53], reduced oxygen consumption in skeletal muscle [54], left ventricular dysfunction in the heart [55], accelerated platelet aggregation [56], and brain cell apoptosis [57].In fact, ER stress has been considered to be a major cause of dopaminergic neuron loss in Parkinson’s disease, and Dahl showed that CDN1163 protected neurons from ER stress-induced cell death in vitro [57]. It has also been recognized that ER stress has an important role in inducing β-cell apoptosis. An adequate store of Ca^2+^ in the ER is essential for maintaining ER function, and the activation of SERCA2 is essential to maintain it. A SERCA2 activator might also improve ER stress, which is induced in T2D (Figure 8). SERCA2 protein expression decreased in various diseases including heart failure. The tissue-targeted overexpression of SERCA2 is a next strategy for the various diseases. SERCA2 activity was regulated with associated proteins (phospholamban, salcolipin, etc.), micro-RNA or post-translational modifications (phosphorylation, SUMOnylation, S-glutathiolation, etc.), which might also be next targets to improve SERCA2 activity [37].

In the present study, there are some limitations and points that should be improved. Firstly, we could not show decreased SERCA2 expression in the liver, heart, and soleus muscle of *db*/*db* mice compared to control mice. In general, *db*/*db* mice are considered a model of systemic ER stress [32,53,58], and SERCA2 dysfunction can be observed in almost all organs. The absence of SERCA2 dysfunction in the liver, heart, and soleus muscle in our study could be due to the relatively young age of the mice. The non-significant difference of VO_2_ in the non-exercise-state experiments might also be due to the relatively young age of the mice. Secondly, though we validated the pharmacological impact of CDN1163 on the skeletal muscle and vascular function, we did not explore the precise molecular mechanism by which SERCA2 activation affected them. We determined the concentration and duration for the administration of CDN1163 according to a previous study [23] and a preliminary study for metabolic effects; however, we did not evaluate dose dependency and long-term effects. Thirdly, we only tested one animal model of diabetes and did not examine the presence of side effects. As CDN1163 has hydrophobic properties, other activators of SERCA2 should be considered for drug development. Further study is required for the assessment of the beneficial effects of SERCA2 activators for the treatment of diabetes.

## 5. Conclusions

An allosteric SERCA2 activator, CDN1163, improved vascular endothelial function, skeletal muscle function, hepatosteatosis, and glucose metabolism in *db*/*db* mice. The activation of SERCA2 is an appealing strategy for the treatment of cardiovascular complications in patients with type II diabetes.

## Data Availability

Data supporting reported results can be obtained from the corresponding author under reasonable request.
